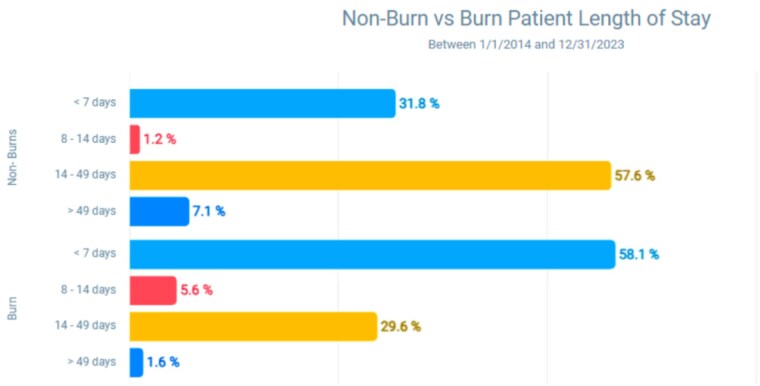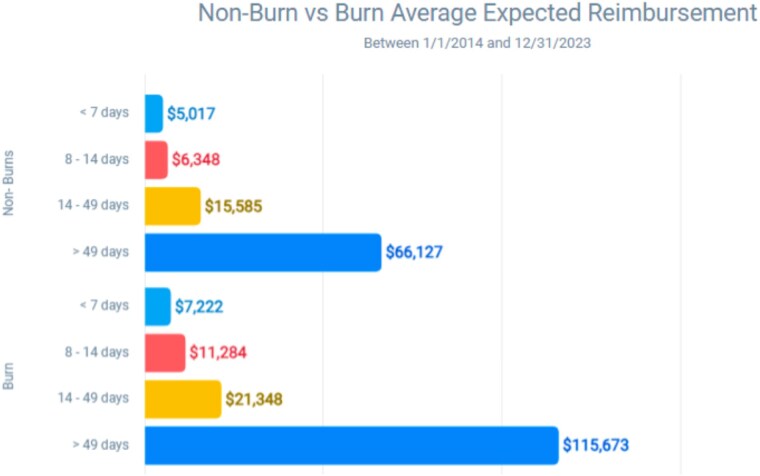# 16 Resource Allocation: Costs and Length of Stay for Non-Burn Patient in Burn Center

**DOI:** 10.1093/jbcr/iraf019.016

**Published:** 2025-04-01

**Authors:** Moon Usman, Alisa Savetamal

**Affiliations:** Connecticut Burn Center, Bridgeport Hospital / Yale-New Haven Health; Connecticut Burn Center, Bridgeport Hospital / Yale-New Haven Health

## Abstract

**Introduction:**

Many burn centers care for patients with skin and soft tissue injury that is not related to burn. For example, skin sloughing exanthems (such as SJS/TEN) and soft tissue pathologies (soft tissue infections, traumatic soft tissue loss) are often referred to a burn center for the expertise in resuscitation and cutaneous care needed by this patient population. In this study, we examine our center’s experience with non-burns in the burn unit, with particular attention to the differences in reimbursement and length of stay when compared with burn patients.

**Methods:**

We conducted a 10-year (2014-2023) retrospective analysis of non-burn patient admissions at a single burn center. A de-identified database was used to stratify patients into burn and non-burn through review of diagnosis codes, and length of stay and expected reimbursement for 2,155 patients (both burn and non-burn) was determined. Four common non-burn etiologies were identified: Stevens-Johnson syndrome/toxic epidermal necrolysis (n = 32), Fournier’s gangrene (n = 8), necrotizing soft tissue infections (n = 31), and frostbite (n = 21). Expected reimbursement was derived from the charges in the electronic medical record. Data were analyzed by patient diagnoses and length of stay to assess the financial and resource implications of treating non-burn conditions within a specialized burn care setting.

**Results:**

Our analysis indicates that non-burn patients have significantly longer hospital stays compared to burn patients. While the majority of non-burn patients (63.7%) stayed over 14 days, the majority of burn patients (58.1%) stayed less than 7 days. Overall, 66% of the non-burn patients required a stay of more than 7 days, whereas only 31% of burn patients needed such an extended stay. At all lengths of stay, the expected reimbursement for non-burns was less than for burns. T-statistic of 7.27 and corresponding p-value < 0.001, reflecting a significant difference between the lengths of stay for non-burn and burn patients. Most strikingly, the average expected reimbursement for non-burn patients with stays over 49 days was $66,127, roughly half the $115,673 average reimbursement for burn patients with similar lengths of stay.

**Conclusions:**

Burn centers demonstrate considerable skill in managing complex non-burn wounds. The expertise in critical care and advanced wound treatment available enhances patient outcomes, albeit at a higher resource cost. These findings highlight the value of burn centers in delivering high-level care for diverse patient populations and suggest a need for balanced resource allocation to optimize care efficiency and cost-effectiveness.

**Applicability of Research to Practice:**

Emphasis the specialized expertise of burn centers in managing non-burn injuries. Insight can efficient resource management and allocating appropriate reimbursement to continue to deliver high-quality treatment for a diverse patient populations.

**Funding for the Study:**

N/A